# Fixed Versus Patient-Specific Trigger Delay in High-Pitch Computed Tomography Angiography of the Aorta Prior to Transcatheter Aortic Valve Implantation: Assessment of Image Quality and Homogeneity of Vessel Enhancement

**DOI:** 10.1097/RLI.0000000000001176

**Published:** 2025-03-21

**Authors:** Sidre Sahin-Uzuner, Foroud Aghapour Zangeneh, Goncalo De Almeida, Oezlem Krzystek, Maria Paslak, Jakob Heimer, Ralf Gutjahr, Thomas Sartoretti, Tilo Niemann, André Euler

**Affiliations:** Department of Radiology, Kantonsspital Baden, affiliated Hospital for Research and Teaching of the Faculty of Medicine of the University of Zurich, Baden, Switzerland (S.S.-U., F.A.Z., G.D.A., O.K., M.P., J.H., T.N., A.E.); Computed Tomography, Siemens Healthineers AG, Forchheim, Germany (R.G.); and Faculty of Medicine, University of Zurich, Zurich, Switzerland (T.S.)

**Keywords:** aorta, computed tomography angiography, contrast media

## Abstract

**Objective:**

The aim of the study is to compare the image quality and homogeneity of vessel enhancement in high-pitch CT-angiography of the aorta (CTA) prior to transcatheter aortic valve implantation between bolus tracking with a fixed trigger delay and bolus tracking with a patient-specific trigger delay.

**Materials and Methods:**

In this retrospective study, consecutive patients who received a CTA of the aorta prior to transcatheter aortic valve implantation between January 2023 and June 2024 were included. Patients were imaged using either bolus tracking and a fixed trigger delay (Group A; 15 seconds) or bolus tracking and a patient-specific trigger delay (Group B; FAST Bolus; Siemens Healthineers AG). The same contrast injection and scan protocol were used in both groups. Vessel enhancement was measured at multiple craniocaudal locations. Subjective image quality was assessed by 2 readers using 5-point Likert scales. Likert scores were analyzed using Wilcoxon rank-sum tests. Enhancement was assessed with a mixed-effects model.

**Results:**

Sixty-five patients (28 females) were assessed in each group. Patient demographics (both 74 ± 12 years; *P* = 0.58, body mass index: 26.0 vs 26.2 kg/m^2^; *P* = 0.79) and radiation dose (CTDI_vol_: 3.4 vs 3.5 mGy; *P* = 0.55) did not differ significantly between the two groups. Mean CT attenuation was 489 HU versus 469 HU in the ascending aorta and 428 HU versus 464 HU in the common femoral artery for fixed and patient-specific delays, respectively. Enhancement in the femoral arteries was significantly lower in the fixed delay group (*P* < 0.05), while there was no significant difference at other vessel locations. Diagnostic image quality and enhancement at the femoral artery were rated significantly better for the patient-specific trigger delay by one reader (both *P* < 0.05).

**Conclusions:**

Bolus tracking with a patient-specific trigger delay improved the craniocaudal homogeneity of vessel enhancement and subjective image quality at the distal access site as compared to bolus tracking with a fixed trigger delay in high-pitch CTA prior to TAVI.

Computed tomography angiography (CTA) is the established imaging modality for evaluating common aortic pathologies, such as aortic aneurysm, aortic dissection, or for preoperative planning prior to transcatheter aortic valve implantation (TAVI).^1–6^ TAVI has been established as an alternative to surgical repair of the aortic valve,^7–10^ which has consecutively increased the use of preoperative planning CTA in the last decade. Ensuring high image quality and uniform contrast enhancement of the vascular lumen throughout the thoracoabdominal aorta is crucial for TAVI planning, enabling precise evaluation of the peripheral interventional access route.^1,11^ Precise scan timing is inevitable for achieving uniform vessel opacification, especially when using high-pitch Computed Tomography (CT) acquisitions. Modern scan protocols primarily rely on bolus tracking or the test bolus technique to optimize the delay between contrast media injection and initiation of the diagnostic scan. In the case of bolus tracking, a predefined fixed trigger delay is applied once a predefined CT attenuation threshold is reached in a monitoring region, such as the ascending or descending aorta. However, contrast dynamics are highly patient-specific, with the cardiac output being the most influential factor.^12^ Variations in cardiac output within a patient population can lead to suboptimal peak arterial enhancement, especially when a fixed scan delay is applied. This is particularly true for high-pitch scans with short scan duration.^13^ In such cases, it becomes even more critical to initiate the scan at the point of maximum contrast enhancement.

In a previous study, Hinzpeter et al^14^ utilized a prototype bolus tracking software with a patient-specific trigger delay for CTA of the aorta. Their findings indicated improved image quality and enhanced contrast media homogeneity in comparison to a fixed trigger delay. However, their studies were limited to a pitch of 1.2, which results in a higher radiation dose compared to a high-pitch acquisition. This software has recently become commercially available for routine clinical practice. To this date, the effect of a patient-specific trigger delay on image quality in high-pitch CTA of the aorta for TAVI planning remains unclear. Therefore, the objective of this study was to compare the image quality and homogeneity of vessel enhancement between bolus tracking with a fixed trigger delay and bolus tracking with a patient-specific trigger delay in high-pitch CTA of the aorta prior to TAVI.

## MATERIALS AND METHODS

### Study Participants

This retrospective single center study was approved by the local ethics committee (BASEC-ID 2024-01290). All participants signed a written informed consent. Consecutive patients ≥18 years of age who underwent a clinically indicated contrast-enhanced, ECG-triggered, high-pitch CTA of the aorta for TAVI planning were screened for inclusion. The screening included cases performed before and after the clinical implementation of the patient-specific bolus tracking software between January 1, 2023 and June 30, 2024. The participants were divided into Group A (bolus tracking with fixed trigger delay) and Group B (bolus tracking with patient-specific trigger delay). Exclusion criteria were defined as denial or an unknown general consent status (n = 78), occlusion or dissection of the thoracoabdominal aorta or iliac vessels (n = 9), aneurysms with intra-aneurysmal contrast retention with consecutive distorted density values (n = 4), artifacts due to inserted total hip arthroplasty or spondylodesis (n = 32) and unknown body height and weight at the time of the examination (n = 2). The primary outcome was CT attenuation of the vessel lumen at multiple craniocaudal locations. The secondary outcomes were homogeneity of vessel lumen enhancement and subjective image quality.

### Imaging Protocol and Data Reconstruction

All CTAs of the aorta prior to TAVI were performed in high-pitch mode on a third-generation dual-source dual-energy CT scanner (SOMATOM Drive, Somaris 7 VB30, Siemens Healthineers AG). The following scan acquisition parameters were applied: systolic ECG triggering, automatic tube voltage selection (CARE kV, slider 11 (optimized for vascular imaging), reference kV of 100), automatic tube current modulation (CARE Dose4D, reference mAs 284), collimation of 128 × 0.6 mm, gantry rotation time of 0.28 s and pitch of 3.2. Axial images with a slice thickness of 1 mm and an increment of 1 mm were reconstructed. The average scan duration in high-pitch mode was 1.3 s. A medium smooth kernel for vascular body examinations (Bv38) and an advanced modeled iterative reconstruction algorithm (ADMIRE) at a strength level of 3 were applied.

The same contrast medium protocol was applied to both groups via the antecubital vein as follows: 10 mL of 0.9%-NaCl, followed by 100 mL of nonionic iodinated contrast media (Iopamiro 370 mgI/mL, Bracco Group, Milan, Italy) and a saline chaser of 50 mL 0.9%-NaCl. The flow rate was 4 mL/s for all injection phases. Bolus tracking was performed by placing a region of interest (ROI) in the ascending aorta at the level of the carina with a trigger threshold of 100 HU at a tube voltage of 100 kV. In the fixed-delay group, our institutional default protocol with a delay of 15 seconds after reaching this threshold was used. In the patient-specific delay group, the scan delay was determined using a novel feature (FAST Bolus, Siemens Healthineers AG), fully integrated into the official scanner software and compatible with the conventional bolus tracking workflow. The software analyzes the measured bolus tracking signal and utilizes a cardiovascular model (a convolution-based prediction of local arterial contrast enhancement) and information about a given contrast injection protocol to adaptively determine the optimal scan timing for each individual patient.

### Assessment of Objective Image Quality

CT attenuation in the vessel lumen was measured by a radiology resident with 2 years of experience by placing ROIs along the entire length of the aorta, iliac, and common femoral vessels at the following locations: ascending aorta at the level of the right pulmonary artery; aortic arch at the origin of the left subclavian artery; descending aorta at the level of the left atrium; abdominal aorta at the origin of the superior mesenteric artery; proximal common iliac artery on both sides; proximal external iliac artery on both sides and common femoral artery on both sides. ROIs were placed within the vessel lumen as large as possible while avoiding the vessel wall and calcifications.

### Assessment of Subjective Image Quality

Two board-certified radiologists [a subspecialized cardiovascular radiologist (F.A.Z.) and a board-certified general radiologist (G.A.)] with 8 and 5 years of experience independently assessed the subjective image quality. The readers were blinded to the trigger method and the CT datasets were presented in a randomized fashion. Image quality was rated using 5-point Likert scales, assessing vessel contrast, contrast homogeneity, contrast at the access route in the common femoral artery and overall diagnostic image quality according to the following scale: 1 (very poor), 2 (poor), 3 (moderate), 4 (good), and 5 (excellent). In addition, image noise was graded as the following: 1 (unacceptable), 2 (above average), 3 (average), 4 (less than average), and 5 (minimal).

### Statistical Analysis

A prior power analysis estimated a required sample size of 64 per group (d = 0.5, alpha = 0.05, power = 0.8). Data distribution was assessed visually, revealing a substantial right skew in the Likert items, which was analyzed using Wilcoxon rank-sum tests to compare Likert scores across categories and readers. Quantitative CT attenuation metrics were analyzed using a linear mixed-effects model with random intercepts for patient identification number. Estimated marginal means and pairwise comparisons between bolus types were calculated without correction for multiple comparisons. All results were considered significant at *P* < 0.05. Continuous variables are presented as mean ± standard deviation (SD), Likert items as median and interquartile range. Patient data was compared using independent *t* tests without correction. Analyses were conducted using the R-software (v4.3.0, R Core Team, Vienna, Austria).

## RESULTS

### Study Population

**FIGURE 1:**
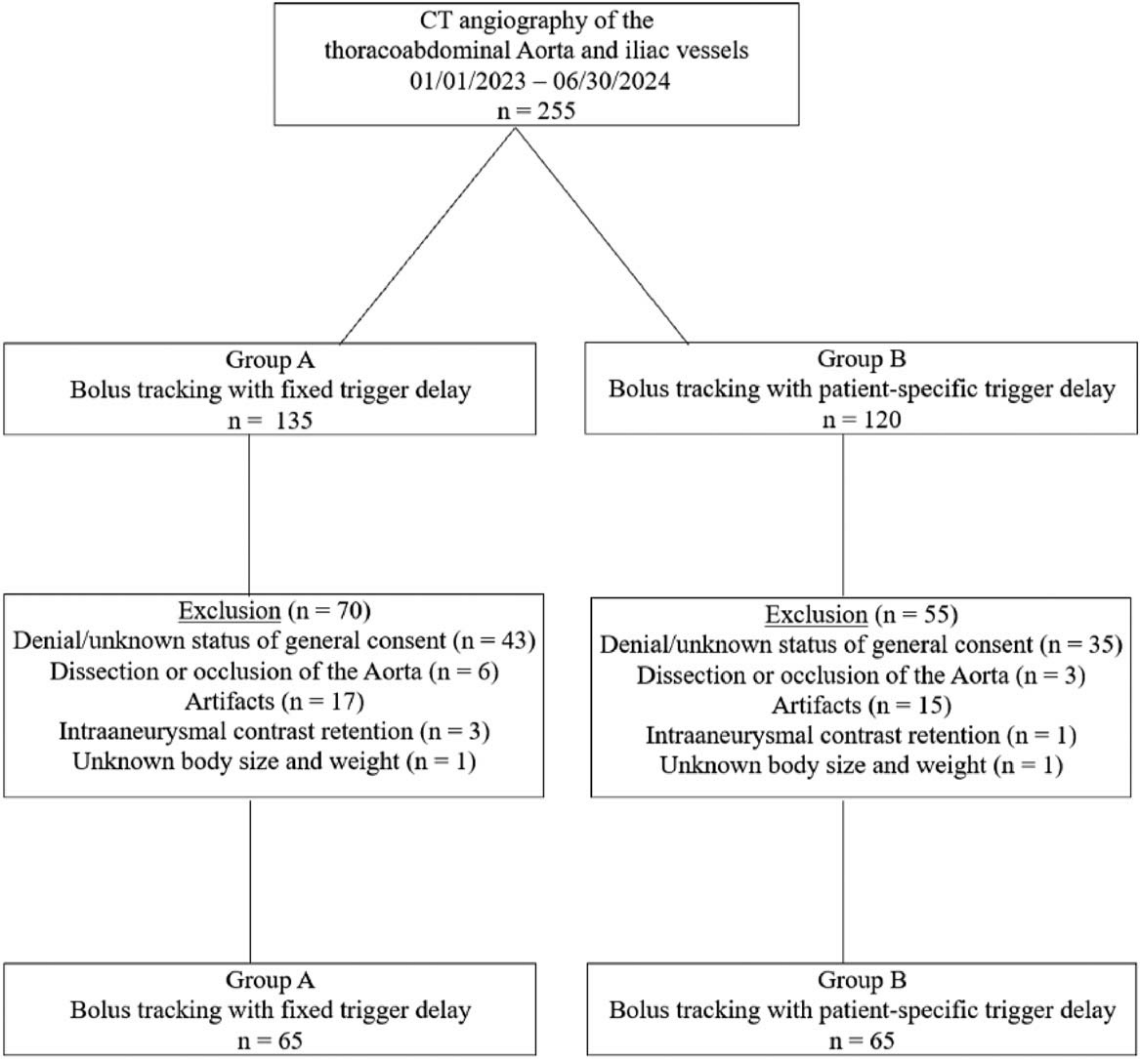
Flowchart of patient inclusion.

Patient selection and characteristics are summarized in Figure 1 and Table 1.

**TABLE 1:** Patient Characteristics and Scan Parameters

	Group A	Group B	*P*
**Patient characteristics**			
**Age (years)**	74 ± 12	74 ± 12	0.58
**Sex**			
**Female, n (%)**	12 (18.5)	16 (24.6)	
**Male, n (%)**	53 (81.5)	49 (75.4)	
**BMI (kg/m** ^**2**^ **)**	26 ± 4.7	26.2 ± 4	0.79
**Heart failure, n (%)**	12 (18.5)	12 (18.5)	1.0
**CAD, n (%)**	27 (41.5)	33 (50.7)	0.38
**Atrial fibrillation, n (%)**	10 (15.4)	14 (21.5)	0.5
**Scan parameters**			
**Delay time (s)**	15	19.5 ± 2.1	
**CTDI**_**vol**_ **(mGy)**	3.4 ± 0.6	3.5 ± 0.9	0.55
**DLP (mGy**×**cm)**	246.4 ± 62.6	250.3 ± 59.9	0.72

Data are means ± standard deviation.

A total of 130 participants (28 females) were included. There was no significant difference in participants age (74 ± 12 years for both groups; *P* = 0.58), body mass index (mean BMI 26.0 vs 26.2 kg/m^2^; *P* = 0.79), coronary artery disease (33 vs 27; *P* = 0.38) or heart failure (12 vs 12; *P* = 1) between the 2 groups. Based on the automatic tube voltage selection, 60 versus 59 patients were imaged at 100 kV and 5 versus 6 patients at 90 kV for Group A and B, respectively. There was no significant difference in radiation dose [volume CT dose index (CTDI_vol_): 3.5 ± 0.9 mGy vs 3.4 ± 0.6 mGy; *P* = 0.55]. The patient-specific trigger delay chosen by the software ranged between 16 s and 27 s with a mean of 19.5 s ± 2.1 s.

### Objective Image Quality

**FIGURE 2:**
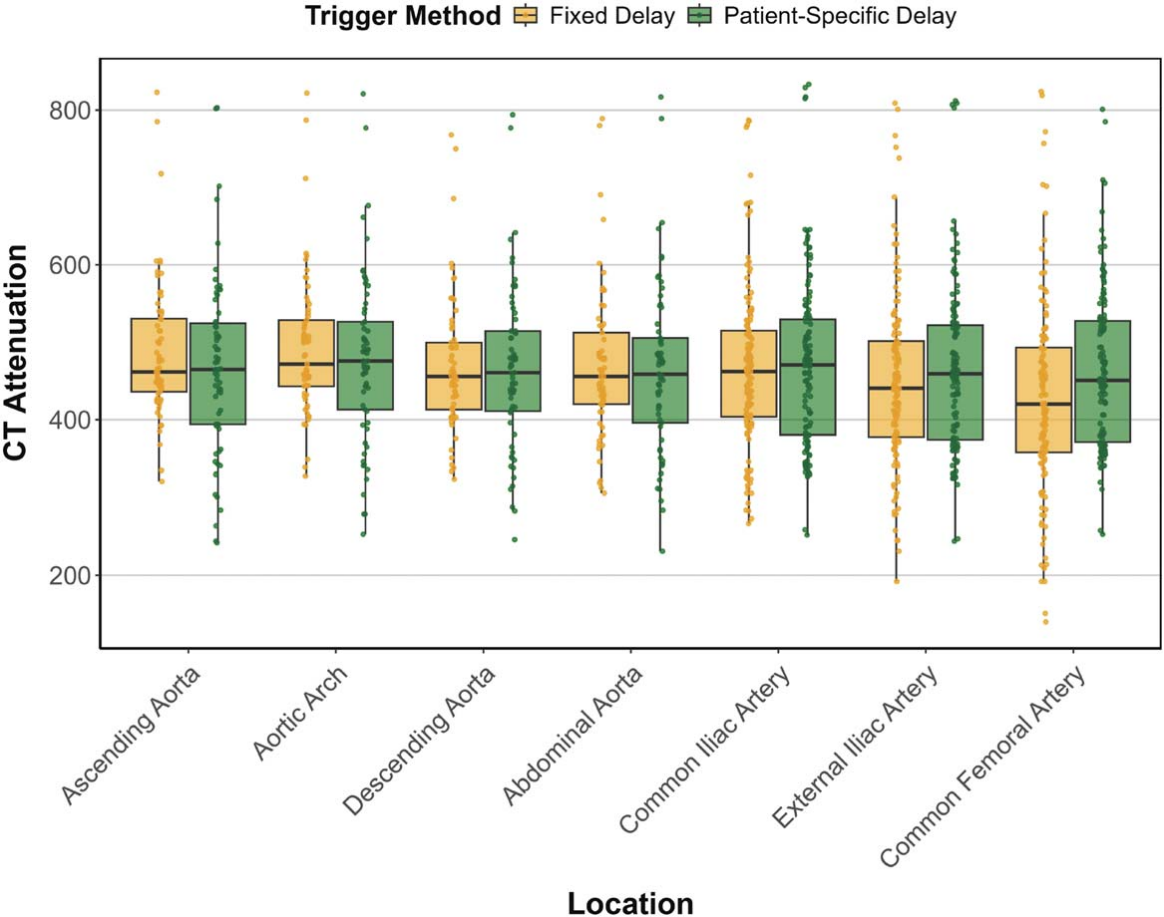
CT attenuation at different craniocaudal vessel locations. Boxplots with mean CT attenuation values on the y-axis and location of measurement of the aorta on the axis in both groups. Note a stable attenuation in group B (patient-specific trigger delay) and lower attenuation in group A (fixed trigger delay) at more distal locations.

**FIGURE 3:**
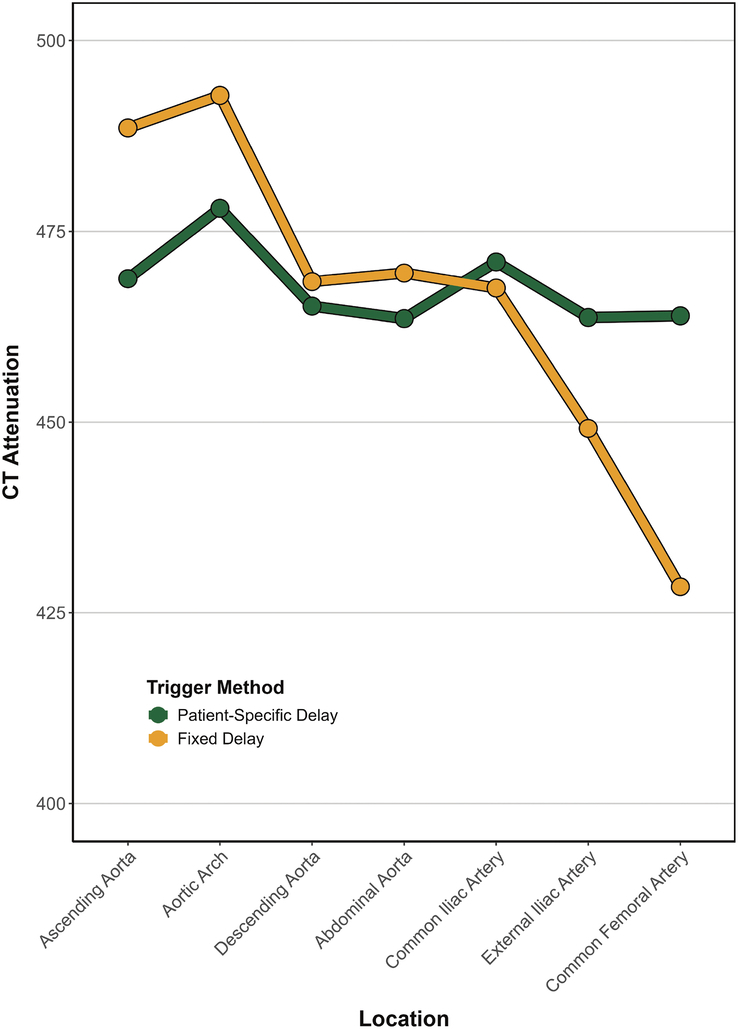
Homogeneity of contrast opacification from proximal to distal locations. Please note the more uniform CT attenuation achieved with the patient-specific trigger delay from cranial to caudal regions of the vascular system. In contrast, the fixed trigger delay resulted in reduced attenuation at the distal vascular access site.

**FIGURE 4:**
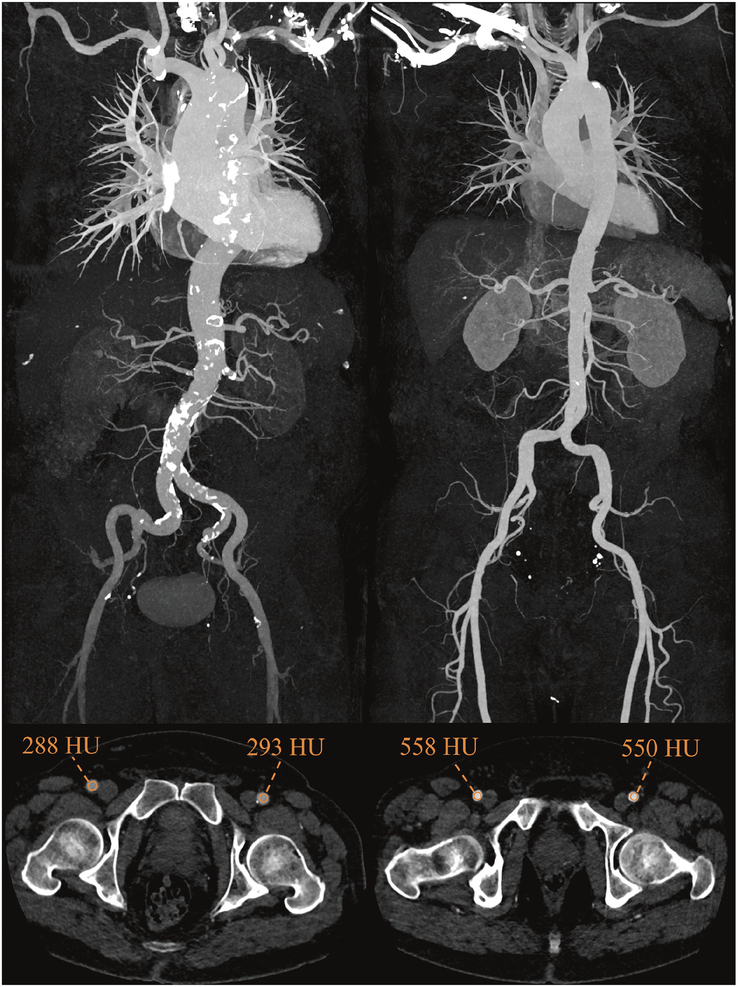
Image example—contrast attenuation along the aorta and at the femoral access site. Coronal maximum intensity projection (top row) and axial CT slice (bottom row) from representative patients imaged with a fixed trigger delay (left) and a patient-specific trigger delay (right) (window width: 800, window center: 430). Note the reduction in enhancement in the craniocaudal direction with the fixed trigger delay compared to the patient-specific trigger delay.

Results are summarized in Table 2 and Table 3, and Figures 2–4.

**TABLE 2:** CT Attenuation at Different Vessel Locations

Measurement Location	Group A	Group B
**Ascending aorta**	489 ± 91	469 ± 115
**Aortic arch**	493 ± 90	478 ± 109
**Descending aorta**	468 ± 88	465 ± 105
**Abdominal aorta**	470 ± 96	464 ± 109
**Common iliac artery**	468 ± 107	471 ± 109
**External iliac artery**	449 ± 113	464 ± 107
**Common femoral artery**	428 ± 126	464 ± 102

*Data are means ± standard deviation in Hounsfield units.

**TABLE 3:** Linear Mixed-Effects Model With post-hoc Pairwise Comparisons of Trigger Methods Across Vessel Locations

Measurement Location	Estimate	T Ratio	*P*
**Ascending aorta**	−19.78	−1.44	0.151
**Aortic arch**	−14.80	−1.08	0.282
**Descending aorta**	−3.22	−0.23	0.815
**Abdominal aorta**	−5.97	−0.43	0.664
**Left common iliac artery**	4.06	0.30	0.768
**Left external iliac artery**	14.35	1.04	0.297
**Left common femoral artery**	34.26	2.49	0.013
**Right common iliac artery**	2.80	0.20	0.839
**Right external iliac artery**	14.66	1.07	0.287
**Right common femoral artery**	36.69	2.67	0.008

Mean CT attenuation was 489 HU versus 469 HU in the ascending aorta and 428 HU versus 464 HU in the common femoral artery for Group A and B, respectively. The linear mixed-effects model evaluated the effects of trigger method and measurement location on CT attenuation. Post hoc comparisons of estimated marginal means revealed that CT attenuation was significantly lower for the fixed trigger delay at the femoral arteries on both sides (L: 34.3, *P* = 0.013; R: 36.7, *P* = 0.008) as compared to the patient-specific trigger delay (Table 3). No significant differences were found at other locations.

### Subjective Image Quality

Results are summarized in Table 4.

**TABLE 4:** Subjective Image Quality

	Reader 1	*P* Value	Reader 2	*P* Value
**Vessel contrast**	5 (4;5) vs 5 (4;5)	0.25	5 (5;5) vs 5 (5;5)	0.10
**Contrast homogeneity**	5 (4;5) vs 5 (4;5)	0.17	5 (4;5) vs 5 (4;5)	0.13
**Contrast at access route**	4 (4;5) vs 5 (4;5)	0.01*	5 (4;5) vs 5 (4;5)	0.22
**Image noise**	4 (4;5) vs 4 (4;5)	0.45	4 (4;5) vs 4 (4;5)	0.35
**Diagnostic image quality**	4 (4;5) vs 5 (4;5)	0.04*	5 (5;5) vs 5 (5;5)	0.21

Unless otherwise specified, data are medians with interquartile range in parentheses. *Statistically significant.

Interobserver agreement, assessed with Cohen's Kappa, revealed low agreement between readers (κ = 0.336, *P* < 0.001). The low level of agreement justified analyzing each reader's data separately. Image noise, vessel contrast and contrast homogeneity did not differ significantly between both groups for both readers (all *P* > 0.05). However, contrast enhancement at the distal access site (common femoral artery) and overall diagnostic image quality were rated significantly higher for the patient-specific trigger delay by 1 reader [4 (4;5) vs 5 (4;5); *P* = 0.012 for enhancement in the femoral artery and 4 (4;5) vs 5 (4;5); *P* = 0.04 for diagnostic image quality, for groups A and B, respectively].

## DISCUSSION

This retrospective study compared objective and subjective image quality, as well as the homogeneity of vessel enhancement between bolus tracking with a fixed trigger delay and patient-specific trigger delay in high-pitch CTA of the aorta performed prior to TAVI. Our findings demonstrated that employing a patient-specific trigger delay significantly improved the craniocaudal homogeneity of vessel enhancement and improved subjective image quality, particularly at the distal access site. This is an important finding considering that the transfemoral approach is the preferred access site for TAVI in approximately 90% of cases.^7,8^ Former studies have shown that the femoral access route is associated with a significantly lower 1-year mortality^15^ and a lower rate of stroke and major life-threatening bleeding compared to a transsubclavian access.^16^ Nevertheless, vascular complications like pseudoaneurysms, dissections, ruptures or arteriovenous fistula formations^17–19^ may occur at a distal access site. Therefore, an optimized and homogeneous contrast enhancement of vessel enhancement in CTA is necessary to select the appropriate access route by considering vessel diameter, calcifications, and tortuosity.^20^

Several studies have focused on optimizing scan and injection protocols for arterial CTA of the aorta.^4–6,11,14,21–25^ These studies have identified several factors that influence image quality, including patient weight, scan parameters, injection flow rate, and injection duration.^21,26^ For instance, Komber et al optimized contrast medium dosing protocols by adjusting contrast medium flux and bolus duration in CTA prior to TAVI, achieving improved opacification at the vascular access site with a 30 s contrast medium bolus delivering an iodine flux of 15- to 19-mg iodine per kg body weight per second.^21^

Beyond these factors, cardiac output can affect aortic peak time and peak enhancement.^27,28^ In our study, we observed a significant trend toward reduced vessel enhancement at more distal vessel locations using a fixed trigger delay. Given that the fixed trigger delay was shorter (15 s) compared to the patient-specific delay (19.5 s ± 2.1 s), we hypothesize that the patient-specific delay was particularly beneficial in patients with a reduced cardiac output. In these patients, a longer time is typically needed to distribute the contrast media to distal vessel locations.

Comparable to our results, a prior study using the prototype of the patient-specific trigger delay demonstrated improved vessel opacification and subjective image quality as compared to a fixed trigger delay in CTA of the aorta.^14^ The authors reported a uniform craniocaudal attenuation of the aorta for the patient-specific trigger delay, while the attenuation decreased at more distal vessel locations for the fixed trigger delay. In contrast to our study, the CTA was performed at a slower pitch value of 1.2, while we investigated a pitch of 3.2. Consecutively, the radiation dose was substantial lower in our study (mean DLP: 382 ± 144 mGy × cm and 358 ± 133 mGy × cm for the fixed and patient-specific delay, respectively in the prior study vs 246 ± 63 mGy × cm and 250 ± 60 mGy × cm for the fixed and patient-specific delay, respectively in our study). Furthermore, the authors utilized shorter fixed (4 s) and patient-specific trigger delays (9.2 s) while employing a smaller contrast media volume of 70 mL. A likely explanation for the shorter delay is that bolus tracking was performed in the descending aorta. In contrast, our institutional TAVI protocol with a fixed delay was already optimized to achieve homogeneous aortic opacification by extending the contrast media bolus and using a fixed delay of 15 s with a contrast media volume of 100 mL. Nonetheless, our findings also demonstrated improved craniocaudal contrast homogeneity with the patient-specific trigger delay.

Bolus tracking with a patient-specific trigger delay has been studied in other vascular regions, including the coronary arteries,^29^ head and neck arteries,^30^ and the abdomen.^31,32^ These studies consistently demonstrated that applying a patient-specific approach can enhance image quality. In the abdomen, Yu et al reported improved image quality and a comparatively adequate subjective image quality for the arterial phase in multiphase abdominal CT.^31^ Similarly, Yuan et al observed higher CT attenuation and improved image quality when using a patient-specific trigger delay in coronary CTA of 102 patients, as well as in head and neck CTA of 196 patients.^29,30^ The benefits regarding contrast homogeneity and improved image quality could also be used to decrease the iodine dose and injection rate as shown in a study by Gutjahr et al in which a patient-specific approach achieved diagnostic image quality despite lower iodine dose.^32^

Our study has several limitations that warrant consideration. First, it was a retrospective, single-center study conducted on a single CT scanner using a standardized contrast media injection and scan protocol. Second, a direct comparison of the 2 bolus tracking methods within the same patient was not feasible, as CTA is typically performed only once prior to TAVI. Third, the fixed-delay cohort utilized a relatively long delay and a larger contrast media volume, as our institutional protocol was already optimized to ensure homogeneous vessel opacification in patients with low cardiac output. We anticipate that greater differences between fixed and patient-specific delays would emerge with lower contrast media volumes and shorter delay times. Fourth, the interreader agreement for subjective image quality was low, highlighting the inherent subjectivity of image quality assessment. Consequently, the 2 scan protocols were analyzed separately for each reader.

In conclusion, bolus tracking with a patient-specific trigger delay improved the craniocaudal homogeneity of vessel enhancement and subjective image quality at the distal access site as compared to bolus tracking with a fixed trigger delay in high-pitch CTA of the aorta prior to TAVI. This could facilitate the preoperative risk assessment regarding the interventional access site. Future studies could focus on the impact of imaging with a patient-specific delay on the planning decision in a prospective randomized-controlled trial.
